# Supplementary material for: Great Enhancement of Carbon Energy Storage through Narrow Pores and Hydrogen-Containing Functional Groups for Aqueous Zn-Ion Hybrid Supercapacitor
Source: Molecules. 2019 Jul 16;24(14):2589. doi: 10.3390/molecules24142589 (PMC6680928; doi:10.3390/molecules24142589)
Supplement: Supplementary file 1 [file molecules-24-02589-s001.pdf]

Supplementary Material for

Great enhancement of carbon energy storage through narrow pores and hydrogen-containing functional groups for aqueous Zn-ion hybrid supercapacitor

Chao Liu [1], Jian-Chun Wu [1], Haitao Zhou [1*], Menghao Liu [1], Dong Zhang [1], Shilin Li [1], Hongquan Gao [1],and Jianhong Yang [1*]

[1] School of Material Science and Engineering, Jiangsu University, 301, Xuefu Road, Zhenjiang, 212013, Jiangsu Province, (P. R. China); haitao19850@ujs.edu.cn

* Correspondence: haitao19850@ujs.edu.cn (H. Zhou); jhyang@ujs.edu.cn (J. Yang)

Figures

Figure. S1

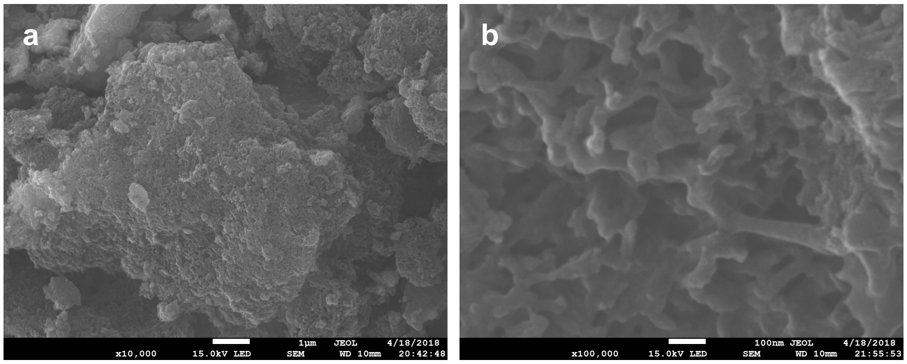

Figure S1. SEM images with low (**a**) and high (**b**) magnifications of the CNSs materials with the bridged and linked fibers structure.

Figure. S2

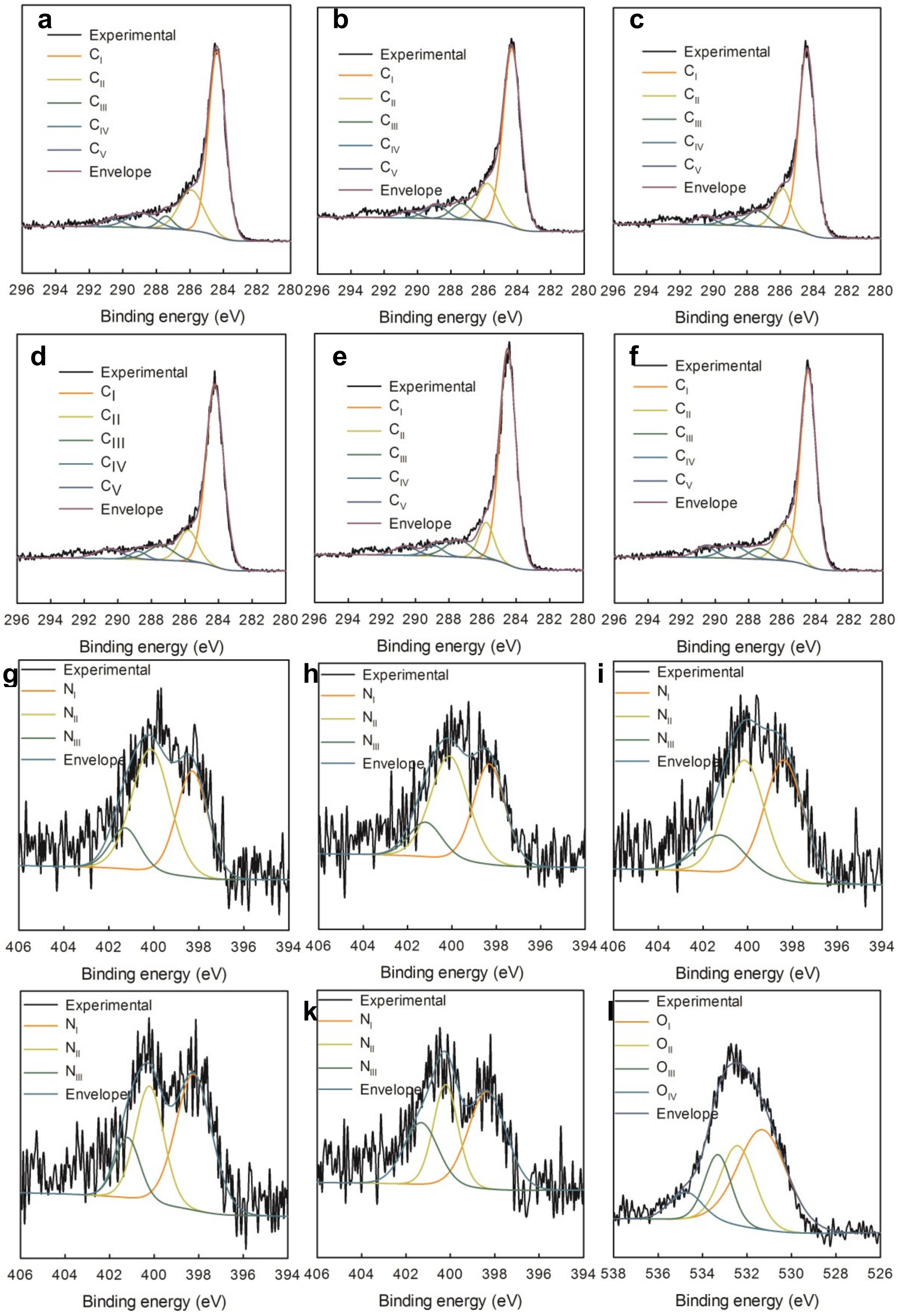

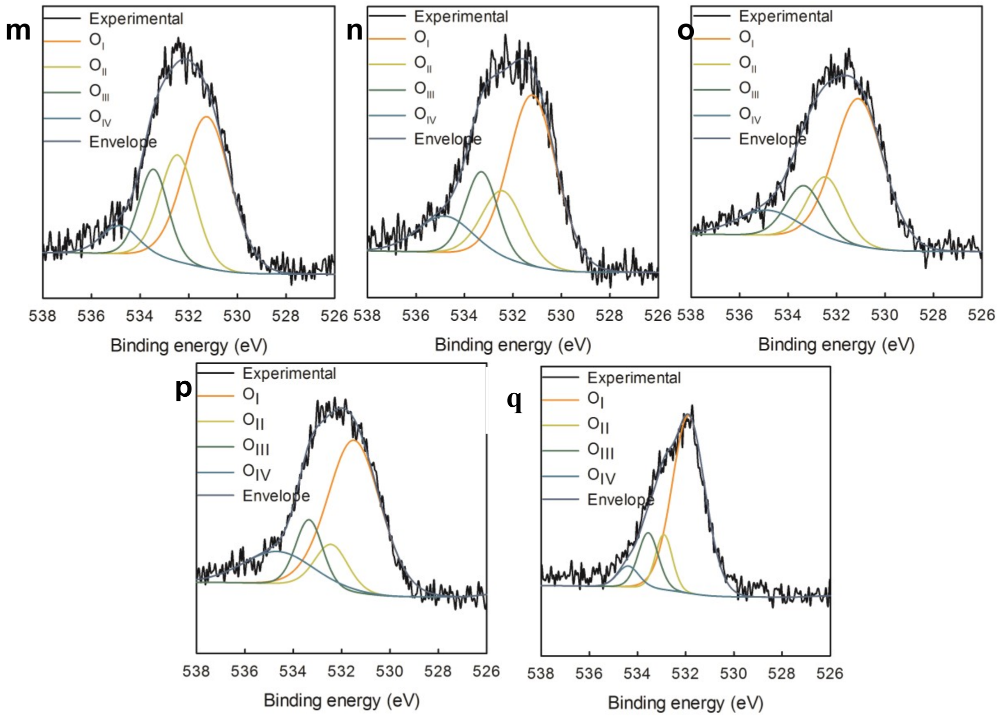

Figure S2. The corresponding C1s, deconvolution of (**a**) FACs, (**b**) FACs200, (**c**) FACs400, (**d**) FACs600, (**e**) FACs800, (**f**) CNSs. The corresponding N1s, deconvolution of (**g**) FACs, (**h**) FACs200, (**i**) FACs400, (**j**) FACs600, (**k**) FACs800. The corresponding O1s, deconvolution of (**l**) FACs, (**m**) FACs200, (**n**) FACs400, (**o**) FACs600, (**p**) FACs800, (**q**) CNSs.

Figure S3

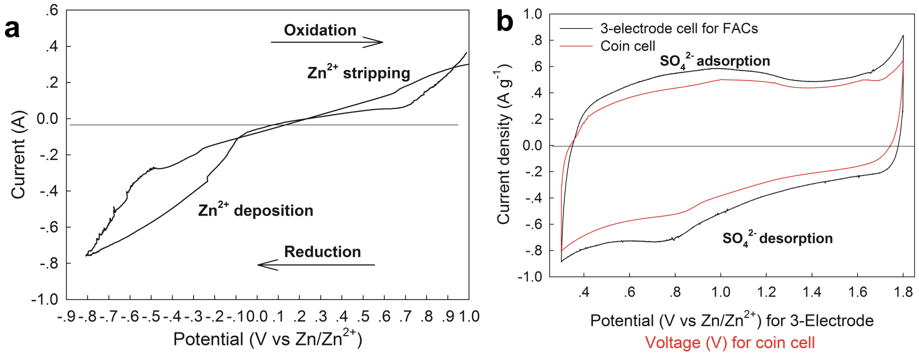

Figure S3. (a) Cyclic voltammetry (CV) curves of the 3-electrode cell obtained using the Zn foil as working electrode, and the FACs cathode as counter electrode at 5 mV s^{-1} and -0.8 to 1 V vs Zn/Zn^{2+} reference. (b) Cyclic voltammetry (CV) curves of the 3-electrode cell obtained using the FACs cathode as the working electrode, and the Zn foil as the counter electrode at 5 mV s^{-1} and 0.3 to 1.8 V vs Zn/Zn^{2+} reference. The CV in the 2-electrode setup using coin cell is also given for comparison.

Figure S4

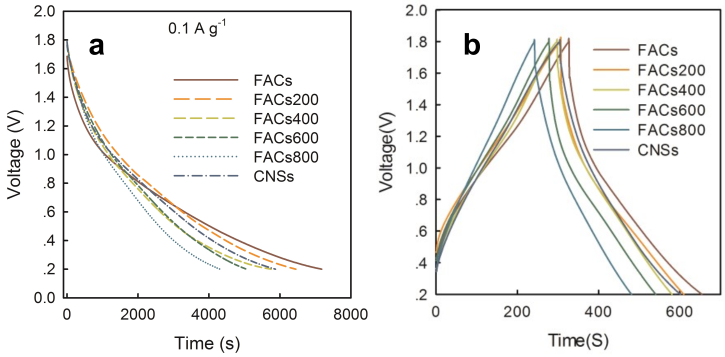

Figure S4 (**a**) Discharge curves at 0.1 A g^{-1} and (**b**) GCD curves of FACs, FACs 200, FACs400, FACs600, FACs800 and CNSs at current densities of 1 Ag^{-1}.

Figure S5

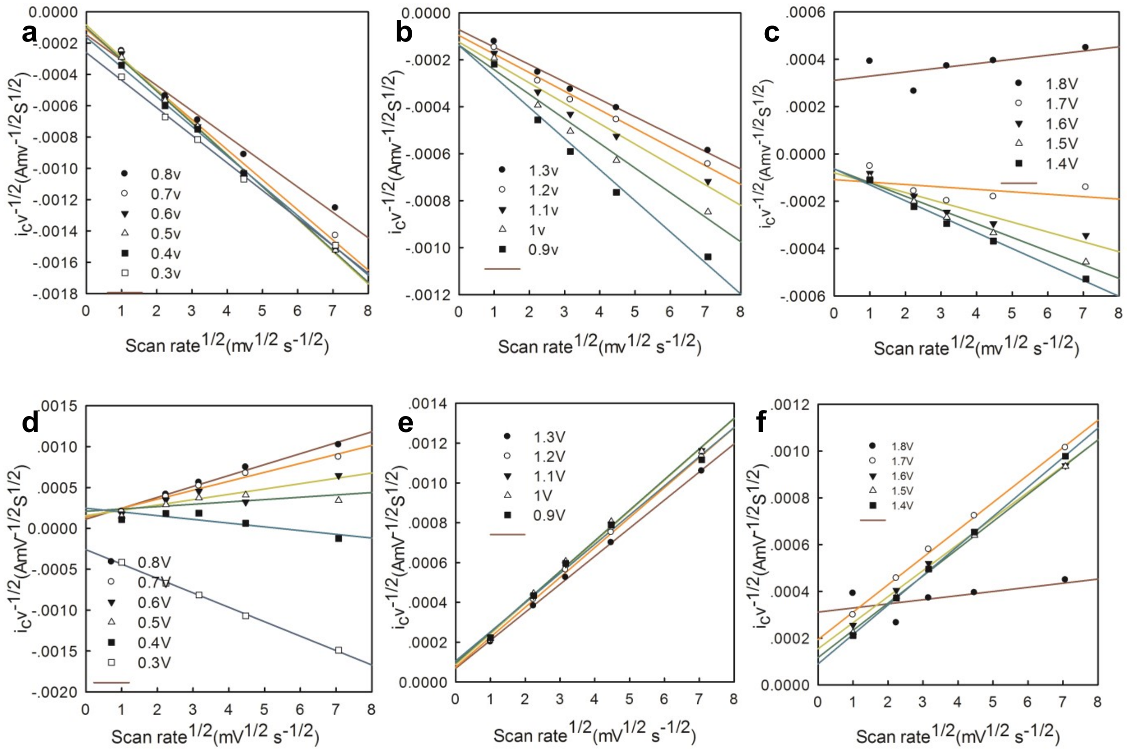

Figure S5. The relationship between the $i_c/v^{1/2}$ and $v^{1/2}$ during the oxidation process **(a)(b)(c)** and reduction process(**d**)(**e**)(**f**).

The relationship between the current (i, in A) and the scan rate (v, in mV s^{-1}) can be expressed as follows.

$$i = av^b \tag{5}$$

In cyclic voltammetry (CV) experiment, the relationship between current and scanning rate is an effective method to distinguish charge storage mechanism. The basis of this evaluation method is that the current of the double layer capacitance process is proportional to the scan rate (v), while the current of the diffusion controlled process is proportional to $v^{1/2}$. When both the diffusion-controlled process and capacitive process coexist during the CV process, the combined current (i_c) can be expressed as shown below [1]:

$$i_c = k_1v + k_2v^{1/2} \tag{6}$$

$$i_c/v^{1/2} = k_1v^{1/2} + k_2 \tag{7}$$

Where k_1 and k_2 are constants for a given potential. The ratio of k_1v and $k_2v^{1/2}$ represents the ratio of capacitive process and diffusion-controlled process. The values of k_1 and k_2 at different potential values are calculated based on the Eq. 6 and 7 (scan rates from 1 mV s^{-1} to 50 mV s^{-1}). The relationship between the $i_c/v^{1/2}$ and $v^{1/2}$ can be plotted during the oxidation (Fig. S5a, b, c) and reduction (Fig. S5d, e, f). The slope and intercept of the line represent the values of k_1 and k_2. Therefore, according to the values of k_1 and k_2 for each potential values, the ratio of capacitive behavior and pseudocapacitive behavior (according to the value of area) was further calculated and drawn in Figure 4e.

Figure S6

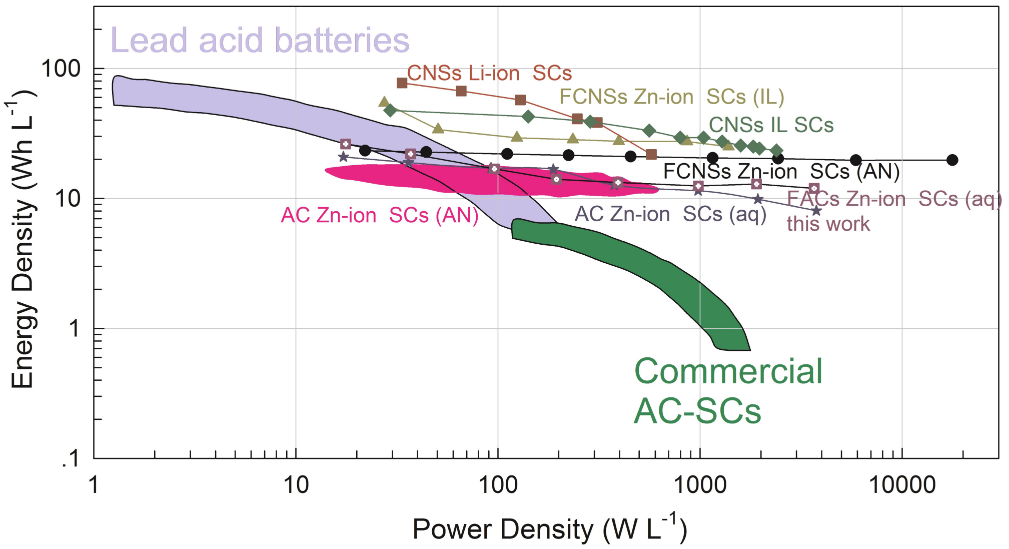

Figure S6. Ragone plot of volumetric energy density vs. power density for Zn-ion hybrid SCs with aqueous electrolyte and FACs in this work (the dark pink squares) and other state-of-art energy storage devices. The compacted density of the FACs electrode is 0.5 kg L^{-1}; the thickness is 100 μm for a commercial SC electrode; the thickness of used stainless steel foil current collector was 20 μm; the air laid paper-based separator was 50 μm; and the commercial available pure Zn foil is 20 μm in thickness. The calculated volume factor was 0.26.FCNSs-based Zn-ion hybrid SCs with both 2.4 V IL and 1.8 V AN electrolytes [2]; the CNSs based Li-ion SCs (4.3V) [3]; the CNSs based symmetric IL SCs (4V) [8]; the AC based Zn-ion hybrid SCs with AN (1.8V) [4]; the AC based Zn-ion hybrid SCs with aqueous electrolyte (1.8 V) [5].

Figure S7

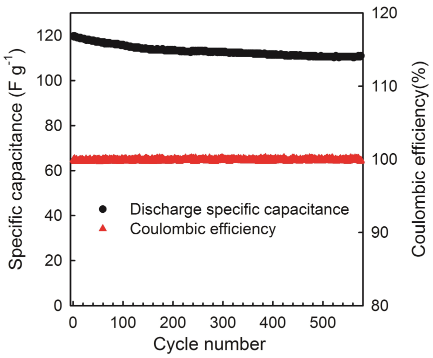

Figure S7 Cycling stability of the FACs based Zn-ion hybrid SC at 2 A g^{-1} for 600 cycles of charge-discharge in a voltage region of 0.3-1.6V.

Figure S8

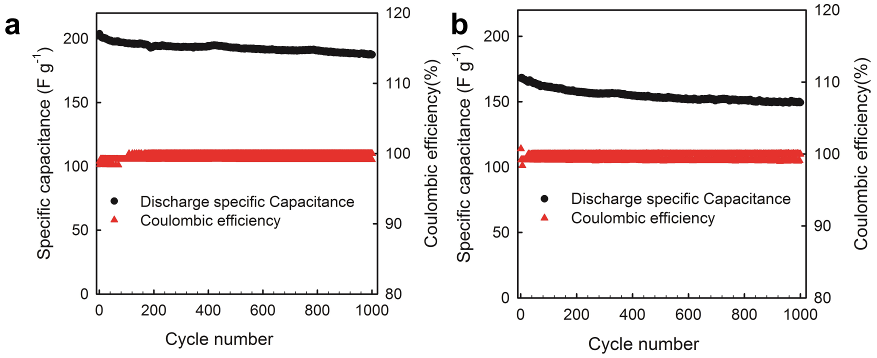

Figure S8 Cycling stability of the FACs based Zn-ion hybrid SCs with electrolyte pH values of 3 (a) and 5 (b) at 2 A g^{-1} for 1000 cycles of charge-discharge in a voltage region of 0.3-1.8V.

Tables

Table S1. Parameters of pores structure calculated from nitrogen adsorption isotherms.

FACs and heat treatment samples

Samples	BET SSA(m^2/g)	BJH mesopores SSA（m^2/g）and ratio(%)	HK micropores SSA ratio（%）	Average pore size (nm)	NLDFT pore volume (cm^3 g^{-1})
FACs	1968.1	135.8(6.9%)	93.1%	1.85	0.82
FACs200	2011.9	61.5(6.9%)	93.1%	1.78	0.89
FACs400	1851.8	61.8(4.51%)	95.5%	1.78	0.83
FACs600	1982.7	96.6(7.27%)	92.7%	1.91	0.95
FACs800	1616.7	78.7(12.5%)	87.5%	1.90	0.77
CNSs	2734.4	808.6(29.6%)	70.4%	2.52	1.61

FACs sample with different activation temperatures

Samples	BET SSA(m^2/g)	BJH mesopores SSA（m^2/g）and ratio(%)	HK micropores SSA ratio（%）	Average pore size (nm)	NLDFT pore volume (cm^3 g^{-1})
650	1545.8	44.6(2.88%)	97.1%	1.80	0.63
700	1968.1	135.8(6.9%)	93.1%	1.85	0.82
750	2040.9	92.0 (4.51%)	95.5%	1.88	0.87
800	2227.0	162.0(7.27%)	92.7%	1.86	0.94
850	2294.9	287.0(12.5%)	87.5%	1.84	0.95
900	3069.8	2111.0(68.8%)	31.2%	3.00	2.15

FCNSs and CNSs sample

Samples H_2O : CO_2：N_2	C	BET SSA(m^2/g)	BJH mesopores SSA（m^2/g）and ratio(%)	HK micropores SSA ratio（%）	Average pore size (nm)	NLDFT pore volume (cm^3 g^{-1})
3：1	0	1237.8	319.0(25.8%)	74.2%	2.29	0.66
4：1	0	1946.7	635.0(32.6%)	67.4%	2.64	1.21
4：1	0.4	2071.9	848.0(40.9%)	59.1%	2.98	1.46
4：1	0.6	2357.7	1070.3(45.4%)	54.6%	3.13	1.77
5：1	0	1925.1	738.0(38.3%)	61.7%	2.87	1.32
CNSs		2734.4	809.0(29.6%)	70.4%	2.53	1.61

Table S2. Summarization of element contents and different functional groups on the surface of FCNSs, FCNSs200, FCNSs400, FCNSs600, and CNSs based on the XPS deconvolution results.

Contents of the elements

Atom type	Atomic content (%)					
	FACs	200	400	600	800	CNSs
C	84.1	84.0	86.0	88.0	88.1	91.5
N	4.0	3.7	4.0	2.8	2.9	1.1
O	11.9	12.3	10.0	9.1	9.0	7.4

Functional groups on the carbon surface

Atom type	Peak label	Peak position/eV	Group	Percentage (%)					
				FACs	200	400	600	800	CNSs
C1s	C_I	284.6	Graphitized carbon	66.6	69.8	71.8	72.7	73.8	67.5
	C_{II}	286.1	C–OH	14.8	13.8	13.0	11.3	8.6	16.5
	C_{III}	287.4	C=O	6.7	7.9	9.0	10.0	11.1	4.5
	C_{IV}	288.9	O=C-O	9.2	5.9	2.6	3.1	3.0	6.4
	C_V	290.6	Carbonate groups	2.7	2.5	3.7	2.9	3.5	5.1
N1s	N_I	398.6	pyridinic N	38.4	38.0	35.9	36.7	36.4	-
	N_{II}	400.5	pyrrolic/pyridone N	40.1	37.9	34.7	30.4	25.2	-
	N_{III}	401.2	quaternary N	21.5	24.2	29.4	32.9	38.4	-
O1s	O_I	531.4	C=O	47.5	49.1	51.7	56.1	59.5	70.5
	O_{II}	532.5	C=O and C–OH	26.6	26.1	18.3	17.7	10.9	12.0
	O_{III}	533.4	C-O-C and C–OH	17.4	17.7	17.3	14.4		13.0
	O_{IV}	534.8	C=O in carboxyl	8.64	7.12	12.77	11.75	13.2	4.6

Reference

1. Zhou, H.; Liu, C.; Wu, J.-C.; Liu, M.; Zhang, D.; Song, H.; Zhang, X.; Gao, H.; Yang, J.; Chen, D., Boosting the electrochemical performance through proton transfer for the Zn-ion hybrid supercapacitor with both ionic liquid and organic electrolytes. *J. Mater. Chem. A* **2019,** 7, (16), 9708-9715.
2. Zhou, H.; Liu, M.; Li, Y.; Liu, C.; Gao, H.; Cao, Z.; Zhang, D.; Jin, X.; Chen, Q.; Liu, Y.; Yang, J., Carbon Nanosponge Cathode Materials and Graphite-Protected Etched Al Foil Anode for Dual-Ion Hybrid Supercapacitor. *J. Electrochem. Soc.* **2018,** 165, (13), A3100-A3107.
3. Wang, X.; Zhou, H.; Lou, F.; Li, Y.; Buan, M. E. M.; Duan, X.; Walmsley, J. C.; Sheridan, E.; Chen, D., Boosted Supercapacitive Energy with High Rate Capability of aCarbon Framework with Hierarchical Pore Structure in an Ionic Liquid. *ChemSusChem* **2016,** 9, (21), 3093-3101.
4. Wang, H.; Wang, M.; Tang, Y., A novel zinc-ion hybrid supercapacitor for long-life and low-cost energy storage applications. *Energy Storage Materials* **2018,** 13, 1-7.
5. Dong, L.; Ma, X.; Li, Y.; Zhao, L.; Liu, W.; Cheng, J.; Xu, C.; Li, B.; Yang, Q.-H.; Kang, F., Extremely safe, high-rate and ultralong-life zinc-ion hybrid supercapacitors. *Energy Storage Materials* **2018,** 13, 96-102.
